# Supplementary material for: The neural influence of autobiographical memory related to the parent-child relationship on psychological health in adulthood
Source: PLoS One. 2020 Apr 13;15(4):e0231592. doi: 10.1371/journal.pone.0231592 (PMC7153857; doi:10.1371/journal.pone.0231592)
Supplement: S1 Table — (DOCX) [file pone.0231592.s001.docx]

**Supplementary Table 1.** The list of situations presented in the experiment.

|  | Positive Situations | Neutral Situations | Negative Situations |
| --- | --- | --- | --- |
| 1 | When I received an award from school | When I confessed my wrongdoings | When I got caught lying |
| 2 | When I did well on a test | When I watched television | When I argued with parents |
| 3 | When I gave them a present | When I stayed still | When I fought with my siblings |
| 4 | When I helped out with work | When I took a rest | When I didn’t keep my promise |
| 5 | When we went on a family trip | When I told them what I need | When they pointed out my mistakes |
| 6 | When we hugged | When I talked about my problems | When I didn’t clean |
| 7 | When I got along with my siblings | When I went out | When I didn’t do my homework |
| 8 | When I cleaned my room | When I expressed my opinion | When I was punished in school |
| 9 | When I ran errands for them | When I picked out clothes | When my room was messy |
| 10 | When I studied | When I was on the phone | When I was late after playing |
